# Mandibular fracture: analysis of 293 patients treated in the Hospital of Clinics, Federal University of Uberlândia

**DOI:** 10.1016/S1808-8694(15)31257-X

**Published:** 2015-10-20

**Authors:** Lucas Gomes Patrocínio, José A. Patrocínio, Bruno Henrique Carrijo Borba, Bruno De Santi Bonatti, Lauro Figueira Pinto, Juliana Villela Vieira, José Mariano Carvalho Costa

**Affiliations:** 1 Resident Physician, Service of Otorhinolaryngology, Federal University of Uberlândia.; 2 Graduate studies in Medicine, Federal University of Uberlândia.; 3 Joint Professor, Head of the Division of Surgery and Buco-Maxillo-Facial Trauma, Service of Otorhinolaryngology, Medical School, Federal University of Uberlândia. Service of Otorhinolaryngology, Medical School, Federal University of Uberlândia, Uberlândia, Minas Gerais.

**Keywords:** mandibular fractures, treatment, complications, etiology

## Abstract

Mandibular fracture is the second most common facial fracture and there has been a significant increase in number of cases in the last years. Misidentification and inadequate treatment can take to permanent aesthetic or functional deformity. **Aim**: Evaluate cases of mandibular fracture reduction in the Hospital of Clinics of the Federal University of Uberlândia, from January of 1974 to December of 2002. **Study design:** historical cohort. **Patient and Method**: Two hundred and ninety-three cases of reduction of mandibular fractures were retrospectively analyzed according to factors related to: patient, trauma, signs and symptoms, and surgical treatment. **Results**: There has been a clear tendency of increase of the number of mandibular fractures along the years. There was higher prevalence in male (4:1), with occurrence peak between 20 to 29 years old. The principal causes of fracture in this study were traffic accidents and violence, representing 72.4%. One hundred and thirty-five patients presented only one fracture. The most injured sites were, in decreasing order, symphysis, condyle, angle, body, ramus, and coronoid. We performed closed reduction (28), open reduction (213) and association of the two (11 patients); 56.8% of the patients were treated within the first 3 days; and, 50.4% were discharged from the hospital until the first postoperative day. About 10% of the patients presented complications, being osteomyelitis the most frequent one. **Conclusion**: The incidence of mandibular fractures was remarkably larger in the male sex, during the third decade of life. The most common cause was traffic accident, and symphysis and condyle were the most injured sites. Isolated fractures occurred in over half of the cases. Most of the patients were treated in the first three days and were discharged until the first postoperative visit. Closed reduction was the treatment most commonly employed. The most frequent complication was osteomyelitis.

## INTRODUCTION

For the past decades, there has been a significant increase in head-maxillo-facial traumas, and mandible fracture occupies the second most frequent incidence of facial bone fractures, with incidence of about 38%. They are mainly caused by car accidents, considering that it is a resistant bone that takes a relatively strong blow to have it fractured, which can also be a consequence of sport activities, firearm or sharp accident, physical assault, work-related accident, metabolic diseases or tumors ^1^.

Given that the mandible is the only facial bone that has mobility and the remaining portion is part of the fixed facial axis, the fracture is never left unnoticed because it is very painful, pain that worsens with mastication and phonation movements, and even respiratory movements; sometimes there are facial asymmetry complaints. Mandible fractures may lead to deformities, be them by displacement or non-restored bone losses, with dental occlusion affection or temporomandible joint disorder (TMJD). If not identified or inappropriately treated, these lesions may lead to severe sequelae, both cosmetic and functional 2, 3.

The present study aimed at assessing 293 patients submitted to mandible fracture reduction at Hospital de Clínicas, Federal University of Uberlândia (HC-UFU), distributed according to gender, age, etiology, location of fractures, treatment techniques and postoperative complications.

## PATIENTS AND METHODS

From January 1974 to December 2002, we performed 293 mandible fracture reductions at HC-UFU, identified through medical chart analysis at the Sector of Infection Control of the institution. We performed an observational epidemiological, descriptive and retrospective study of the medical charts of these cases.

Generally speaking, the collected information from the medical charts were: patients’ data (age, gender), trauma (date, etiology, fracture site, associated lesions), clinical picture and surgical treatment (technique, reduction date, length of stay, complications).

Clustered data were analyzed by Microsoft Access 2000^®^ software and based on it, simple frequencies and results were converted into Microsoft Excel2000^®^ charts and Microsoft Word 2000^®^ tables.

The present study was approved by the Research Ethics Committee of the institution.

## RESULTS

**Graph 1:**
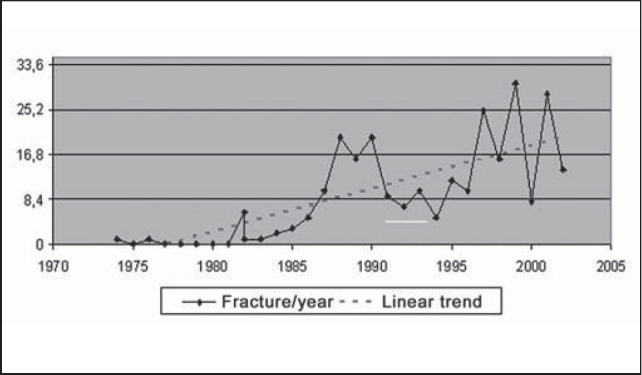
Evolution of number of mandible fracture reductions per year, HC-UFU, 1974-2002.

**Graph 2:**
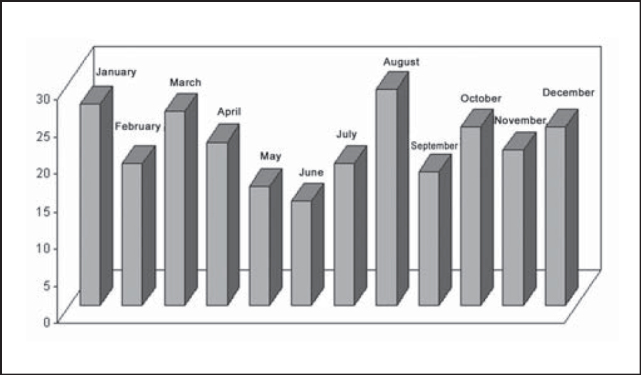
Distribution of mandible fracture reductions according to month of occurrence, HC-UFU, 1974-2002.

In the studied period, we observed a clear trend of increase in number of mandible fractures throughout the years (Graph 1). However, we did not detect significant variations between number of fractures and months of occurrence, but the months that had the smallest number of occurrences on average were May (16), September (18), July and February (19 cases). August, January and March were the months with highest incidence, with 29, 27 and 26 cases on average (Graph 2).

**Graph 3:**
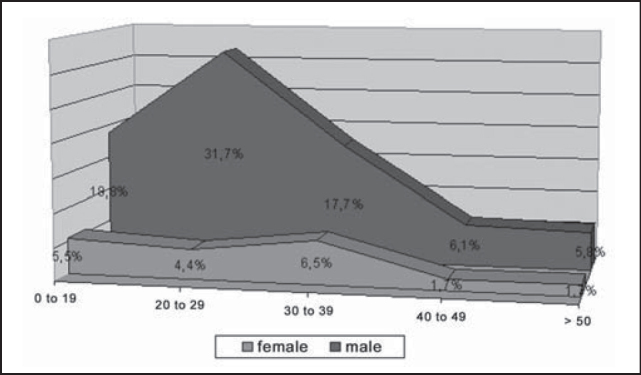
Distribution of mandible fracture reductions according to gender and age range, HC-UFU, 1974-2002.

By crosschecking the data collected from age and gender, we detected a predominance of male gender cases in all age ranges, mean of approximately 4:1. We also detected a peak of occurrence in young adults, aged 20 to 29 years (Graph 3).

The main causes of mandible fractures in the study were traffic accidents (143 cases) and violence (69 cases, 9 injuries due to firearm), amounting to 72.4% of the total (Table 1).

**Table 1:** Distribution of mandible fracture reductions according to etiology, HC-UFU, 1974-2002.

Etiology	N absolute	% total
Traffic accident	143	48.8
Interpersonal violence	69	23.5
Falls	34	11.6
Sport and leisure	15	5.1
Work-related	9	3.1
Non-identified	23	7.9

Total	293	100%

The clinical presentation of patients when they first arrived at HC-UFU comprised specific signs and symptoms of trauma (pain and local edema). Next, we found specific signs and symptoms of mandible fracture (difficulty to open the mouth, abnormal movements, malocclusion).

**Figure 1:**
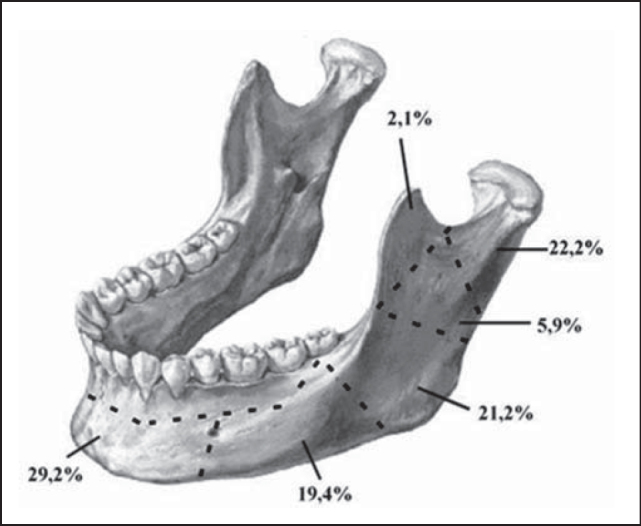
Schematic representation of the mandible fracture reductions according to location, HC-UFU, 1974-2002.

Among the analyzed patients, 135 (64.3%) presented one single mandible fracture, 71 (33.8%) had 2 and 4 (1.9%) had three fractures. The most affected sites were, in decreasing order, symphysis, condyle, angle, body, ramus and, finally, the coronoid process (Figure 1). In 83 medical charts, there were no references to fracture site. In sixty patients (10.5%), there was open fracture.

One hundred and forty-two patients (48.5% of the total) presented pathologies associated with mandible fracture. Out of the total, the most frequent were orthopedic fractures (33), maxilla fractures (25), zygoma fractures (18), oral cavity organ damage (13), thoracic trauma (12), orbit fractures (11) and nasal fractures (8 cases).

**Graph 4:**
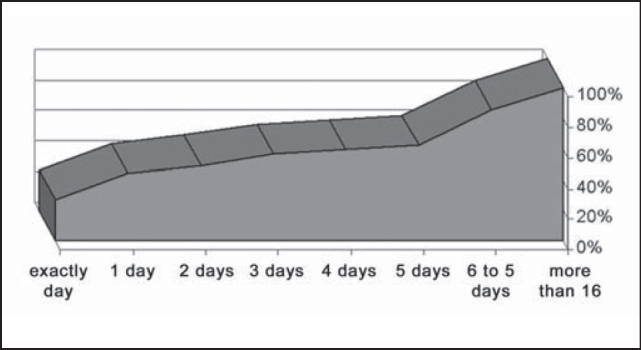
Distribution of number of days after the trauma up to mandible fracture reduction, HC-UFU, 1974-2002.

**Graph 5:**
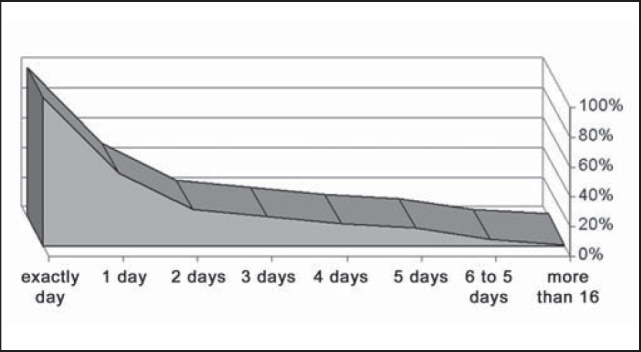
Distribution of number of days after the trauma up to hospital discharge, HC-UFU, 1974-2002.

As to the period from fracture occurrence to treatment day, 26.8% of the patients were treated on the same day, 56.8% were treated within the first 3 days, and 73.2% were treated within the first week (Graph 4). A total of 35 patients (14.0%) were submitted to treatment only after 15 days or more. The type of reduction used was closed (28 patients), open (213 patients) and their combination (11 patients). The time until discharge ranged a lot, depending on patients’ conditions and severity of associated lesions, ranging from the same day to 82 days after. Most patients (50.4%) were discharged in the early postoperative period and only 4.7% remained in the hospital for over 5 days (Graph 5).

About 10% of the patients presented complications resulting from the mandible fracture and its subsequent treatment, and 4 patients presented more than one complication (Table 2).

**Table 2:** Distribution of mandible fracture reductions according to complications, HC-UFU, 1974-2002.

Complications	N absolute	% total
Osteomyelitis	18	6.1
Pseudoarthrosis	6	2.0
Partial function loss	5	1.7
Neural damage	2	0.7
Total function loss	1	0.3

Total	32	10.9

## DISCUSSION

The mandible is the only mobile bone of the face and it participates in basic functions such as mastication, phonation, swallowing and maintenance of dental occlusion ^2^.

Despite the fact that it is the heaviest and strongest facial bone, the mandible is prone to fractures for some specific reasons: 1) it is an open arch; 2) it is located in the lower portion of the face; 3) it is the mechanism of hyperextension and hyperflection of the head in traffic accidents; 4) it gets atrophy as a result of aging ^4^.

In this historical series, we evidenced the linear trend of growth of the incidence of mandible fractures treated at HC-UFU. It is justified by the increased demographics in the region, associated with improved healthcare services and access to the services. In addition, there has been an increase in number of traffic accidents and interpersonal violence incidents 5, 6, 7.

Data related with gender demonstrates male over female predominance in a rate of 4:1, which is in agreement with the literature 2, 5, 7, 8, 9, 10. The age range 20 to 29 years was the most affected one, a fact that coincides with data from mandible fractures 7, 9, but also from other facial bones 11, 12. The predominance of male gender in the age range 20-29 years is due to the fact that this group is more prone to traffic accidents and violence, normally associated with use of alcoholic beverage 6, 7, 10.

There are two basic etiologies for mandible fractures. We name pathological fractures the ones related with tumors, osteoporosis and other diseases that directly or indirectly affect the bone. Traumatic fractures are the most frequent mandible fractures and are related to traffic accidents, falls, violence, sport activities, among others.

The causes of fracture have extremely variable incidence depending on social, geographical and economic characteristics ^6^. In the present study, the decreasing order of frequency found was: traffic accidents, aggression and/or violence, falls, sport; leisure and finally, work-related fractures. These data are in agreement with the current literature 7, 11, 14, 15. There is an increasing trend in fractures caused by traffic accidents and violence owing to the current epidemiological morbi-mortality profile, basically an urban movement ^7^. There are countries whose main cause of mandible fracture is related with sport activities, such as in Austria ^16^. Others present interpersonal violence as the most common cause, such as Hawaii^17^, Zimbabwe^18^, Canada ^19^. It is important to point out that there is predominance of occurrence of fracture at night hours and early Saturday and Sunday mornings, related with higher intake of alcoholic drinks 6, 10.

The clinical history is extremely important for the diagnosis of mandible fractures. We classically find a set of signs and symptoms comprising pain, edema, hematoma, dental desocclusion, facial contour deficit, cracking, and mobility of bone fragments. The main radiological incidences are AP for the mandible, facial absolute profile with open mouth, right and left oblique lateral incidence for mandible and Towne^6^.

The mandible fracture site is variable, depending on the many different causes of the fracture. Therefore, the literature differs a lot concerning the affected sites. In the present study, the symphysis and condyle were the most affected. Symphysis and parasymphysis fractures are also the most common in studies conducted in Singapore, amounting to 46.5% of the cases ^20^. Condyle fractures result mainly from traffic accidents and falls; assault and firearm incidents cause fracture of the mandibular body ^7^.

The percentage of single mandible fractures (64.3%) coincides with other mandible fracture indexes reported in large centers 2, 7, 9. In sixty patients (20.5%), there was occurrence of open fracture, whose data coincided with the world literature owing to the fact that mandible fractures are normally caused by major impact trauma (traffic accident, sport, firearm and others) 2, 7, 9. Cranio-maxillo-facial and other system traumas have also been concomitantly detected, in agreement with the literature 15, 17.

There are many different therapeutic possibilities, given that many authors disagree about the best treatment approach. Regardless of the type of fracture and treatment, we should achieve anatomical reduction by positioning the teeth and precisely readjusting bone fragments for appropriate treatment, whose main objective is to maintain mandible function ^21^. Therefore, the objectives of the therapy are: mandible symmetry, absence of pain or cracking upon TMJ palpation with closed and opened mouth, satisfactory dental occlusion, maximum interincisal opening greater than 40mm, and absence of midline deviation or deviation smaller than 2mm at mouth opening ^22^.

At HC-UFU, mandible fractures were treated with two basic forms of treatment, either open or closed approach. Part of single and simple fractures of the mandible can receive closed treatment. Closed treatment should be preferred in cases of single, simple or bilateral fractures, with little deviation, when the number of teeth and dental support provide conditions for the stability of the occlusion. Symphysis, parasymphysis and body fractures with some vertical and horizontal deviations can be treated with closed approach ^1^. In the present study, 39 fractures were submitted to closed reduction.

The open treatment is formally indicated in the following situations:

• Mandible angle fracture.
• Fracture with deviation of symphysis and parasymphysis region.
• Fracture in edentulous patients.
• Communitive fracture and instability.
• Associated fracture that produces significant deviation.
• Fracture with mechanical interposition upon reduction with incarceration of muscle or teeth.
• Fracture associated with midface disjunction.
• Pathological fracture or fracture associated with deciduous teeth.
• In cases in which closed treatment did not manage to reach bone alignment or maintenance of this situation.

In the present study, 224 fractures were submitted to open reduction, which is in agreement with other literature studies 15, 20, 21, 23.

The surgery based on the principle of reduction and fixation of bone fragments should be conducted as early as possible and as soon as clinical conditions allow it, because most of the patients have to be submitted to general anesthesia, with nasotracheal intubation preferably ^1^. In the present study, 26.8% of the patients were treated on the same day, 56.8% within the first 3 days, and 73.2% within the first week, that is, there was confirmation of the need for early treatment.

We reached a postoperative complication rate of 10.9%, compatible with literature data 3, 7, 19, 24, 25. The rate of postoperative infection was 6.1%, and it was favorably comparable to many different series in the literature, which varied from 0.5 to 22% ^22^. Most patients (50.4%) were discharged right at the early postoperative visit, confirming the low rate of complications. A minority of patients (4.7%) remained in the hospital for more than 5 days. Normally, it was a result of comorbidities.

## CONCLUSIONS

In the present study, the incidence of mandible fractures was more prevalent in male patients, especially during the third decade of life. The most common cause was traffic accident and the more frequently affected regions were symphysis and condyle. Isolated mandible fractures occurred in more than half of the cases. Most patients were treated within the first three days and were discharged right after the early postoperative visit. Open reduction was the most commonly used treatment. The most frequent complication was osteomyelitis.
